# Clinical and Genetic Features in Patients With Reflex Bathing Epilepsy

**DOI:** 10.1212/WNL.0000000000012298

**Published:** 2021-08-10

**Authors:** Andrea Accogli, Gert Wiegand, Marcello Scala, Caterina Cerminara, Michele Iacomino, Antonella Riva, Barbara Carlini, Letizia Camerota, Vincenzo Belcastro, Paolo Prontera, Alberto Fernández-Jaén, Nerses Bebek, Paolo Scudieri, Simona Baldassari, Vincenzo Salpietro, Giuseppe Novelli, Chiara De Luca, Celina von Stülpnagel, Felicitas Kluger, Gerhard Josef Kluger, Gabriele Christine Wohlrab, Georgia Ramantani, David Lewis-Smith, Rhys H. Thomas, Ming Lai, Alberto Verrotti, Salvatore Striano, Christel Depienne, Carlo Minetti, Fabio Benfenati, Francesco Brancati, Federico Zara, Pasquale Striano

**Affiliations:** From IRCCS Istituto Giannina Gaslini (A.A., M.S., M.I., A.R., B.C., P.S., S.B., V.D.S., C.M., F.Z., P.S.); Department of Neurosciences, Rehabilitation, Ophthalmology, Genetics, Maternal and Child Health (DINOGMI) (A.A., M.S., P.S., V.D.S., C.M., F.Z., P.S.), University of Genoa, Italy; Neuropediatrics Section of the Department of Pediatrics (G.W.), Asklepios Clinic Hamburg Nord-Heidberg, Hamburg; Department of Pediatric and Adolescent Medicine II (Neuropediatrics, Social Pediatrics) (G.W.), University Medical Centre Schleswig-Holstein, Kiel, Germany; Department of Neurosciences (C.C., C.D.L.), Pediatric Neurology Unit, Tor Vergata University, Roma; Human Genetics (L.C., F. Brancati), Department of Life, Health, and Environmental Sciences, and Department of Pediatrics (A.V.), University of L'Aquila; Child Neuropsychiatry Unit (V.B.), Department of Mental Health, ASST-LARIANA, Como; Medical Genetics Unit (P.P.), "S. Maria della Misericordia" Hospital, Perugia, Italy; Department of Pediatric Neurology (A.F.-J.), Hospital Universitario Quirónsalud and Universidad Europea de Madrid, Madrid, Spain; Istanbul University Istanbul Faculty of Medicine (N.B.), Department of Neurology, Turkey; Department of Biomedicine and Prevention (G.N.), Tor Vergata University of Rome; IRCCS Neuromed (G.N.), Pozzilli, Italy; Department of Pharmacology (G.N.), School of Medicine, University of Nevada, Reno; Department of Pediatrics (C.v.S.), University Hospital Munich, Germany; Paracelsus Medical University (C.v.S.), Salzburg, Austria; Epilepsy Center for Children and Adolescents (F.K., G.J.K.), Vogtareuth, Germany; Department of Neuropediatrics (G.C.W., G.R.), University Children's Hospital Zurich, Switzerland; Translational and Clinical Research Institute (D.L.-S., R.H.T., M.L.), Newcastle University; Department of Clinical Neurosciences (D.L.-S., R.H.T., M.L.), Newcastle Upon Tyne Hospitals National Health Service Foundation Trust, UK; Epilepsy Center (S.S.), Federico II University, Napoli, Italy; Institute of Human Genetics (C.D.), University Hospital Essen, University of Duisburg-Essen, Essen, Germany; Institut du Cerveau et de la Moelle épinière (ICM) (C.D.), Sorbonne Université, UMR S 1127, Inserm U1127, CNRS UMR 7225, Paris, France; Center for Synaptic Neuroscience and Technology (F.Benfenati), Istituto Italiano di Tecnologia; IRCCS Ospedale Policlinico San Martino (F. Benfenati), Genoa; and Human Functional Genomics (F. Brancati), IRCCS San Raffaele Pisana, Rome, Italy

## Abstract

**Objective:**

To describe the clinical and genetic findings in a cohort of individuals with bathing epilepsy, a rare form of reflex epilepsy.

**Methods:**

We investigated by Sanger and targeted resequencing the *SYN1* gene in 12 individuals from 10 different families presenting with seizures triggered primarily by bathing or showering. An additional 12 individuals with hot-water epilepsy were also screened.

**Results:**

In all families with bathing epilepsy, we identified 8 distinct pathogenic or likely pathogenic variants and 2 variants of unknown significance in *SYN1*, 9 of which are novel. Conversely, none of the individuals with hot-water epilepsy displayed *SYN1* variants. In mutated individuals, seizures were typically triggered by showering or bathing regardless of the water temperature. Additional triggers included fingernail clipping, haircutting, or watching someone take a shower. Unprovoked seizures and a variable degree of developmental delay were also common.

**Conclusion:**

Bathing epilepsy is genetically distinct reflex epilepsy caused mainly by *SYN1* mutations.

Reflex epilepsies (REs) refer to conditions characterized by recurrent seizures triggered primarily by specific motor, sensory, or cognitive stimulation.^1,2^ Acquired or genetic etiologic factors are believed to underlie complex and largely unknown pathophysiologic mechanisms that ultimately lead to hyperexcitability of cortical or subcortical neuronal areas in response to physiologic stimuli.^3^ The genetic background of REs is highly heterogeneous, and only a few causative genes have been identified in humans.^4-7^

Hot-water epilepsy (HWE) and bathing epilepsy (BE) are among the most common REs in the pediatric population^8,9^ and are considered part of the same spectrum.^10^ However, recent evidence suggests that they are different entities with distinct genetics, triggers (hot vs pouring water), clinical presentation, and comorbid conditions. Indeed, autosomal dominant pedigree with reduced penetrance allowed mapping 2 loci for HWE at chromosomes 10q21.3-q22.3^11^ and 4q24-q28,^12,^ and affected children have otherwise normal development. Conversely, BE has been associated with mutations in the X-linked gene *SYN1* that encodes 1 of the 3 Synapsins (SYN1–SYN3), a family of phosphoproteins involved in synaptic development, function, and plasticity.^13,14^ In addition to seizures triggered by water, *SYN1* mutations are responsible for a wide range of neurodevelopmental disorders, including cognitive impairment, autism spectrum disorders (ASD), and unprovoked seizures.^15,16^ To date, *SYN1* mutations have been described in 9 patients with BE^17-19^ and 1 patient with HWE.^20^

We report the clinical and genetic findings of 12 individuals from 10 unrelated families affected by BE, all bearing variants in *SYN1*. The comprehensive analysis of our large cohort and additional cases reported in the literature indicate that BE is a genetically homogeneous distinct RE having SYN1 as its major causative gene.

## Methods

### Study Design and Participant Recruitment

**Figure 1:**
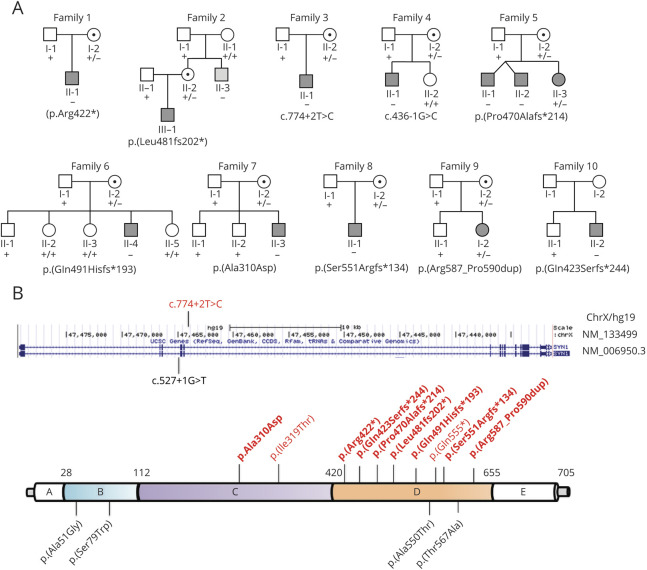
Pedigrees and *SYN1* Mutations of Affected Patients (A) Pedigree of the 10 families showing affected members with bathing epilepsy (BE) (shaded in gray) and healthy female carriers (indicated with a central dot); the uncle of family 2 (II:3) with unprovoked seizure is shown in light gray. (B) Nonsense, frameshift, and missense variants in *SYN1* (NM_006950.3) are depicted along with the Synapsin-1 structure (bottom; protein domains: A; B, linker; C, actin-binding and synaptic-vesicle binding; D, Pro-rich linker; E), and splicing variants are displayed along the genomic locus (top). *SYN1* variants related to BE are in red, and those linked to other clinical presentations than BE are in black. Variants identified in our cohort with BE are in bold.

We enrolled 21 previously unreported probands from 10 unrelated families (figure 1A) with RE induced by showering or bathing through the Network Therapy of Rare Epilepsies. We included patients with bathing/showering-induced seizures documented via either video-EEG or home video recordings by the parents. Clinical data, including genetic findings, neurodevelopmental performance, epilepsy phenotype, and treatment response, were collected with an anonymized, electronic questionnaire. Interictal/ictal (video)-EEG recordings, brain MRI, and neuropsychological tests were centrally reviewed. The neuropsychological and behavioral evaluation was assessed by the Wechsler Intelligence Scale for Children-IV, Wechsler Preschool and Primary Scale of Intelligence-III, Autism Diagnostic Observation Schedule, and Griffiths Mental Development Scale–Extended Revised.

### Genetic Investigations

Genomic DNA was isolated from leukocytes of peripheral blood by the use of standard protocols. Target genetic analysis of *SYN1* was performed by Sanger sequencing in individuals of families 1 through 4, 7, and 8. Other individuals were investigated either by epilepsy gene panels (families 6, 10) or whole-exome sequencing (families 5, 9), and identified variants were confirmed by Sanger sequencing (additional details about sequencing process and data analysis are available in supplemental data, doi.org/10.5061/dryad.w0vt4b8qr). Variants were classified according to the American College of Medical Genetics and Genomics guidelines.^21^ In parallel, we screened for *SYN1* mutations in a previously reported^10^ cohort of 21 patients with HWE to gain further insights into genotype-phenotype correlations of water-related REs.

### Standard Protocol Approvals, Registrations, and Patient Consents

Ethics approval from the IRCCS “G. Gaslini” Institute (Genova, Italy) was obtained for this study. We received written informed consent from all patients (or guardians of patients) participating in this study and authorization for disclosure (consent-to-disclose) of any recognizable persons in photographs and videos.

### Data Availability

Supplemental data, including clinical descriptions, methods of genetic testing, EEGs, and tables of previously reported cases with BE and HWE, are available on Dryad (doi.org/10.5061/dryad.w0vt4b8qr). Videos are available on the *Neurology*® website.

Additional anonymized data that support the findings of this study are available from the corresponding author (P.S.) on reasonable request. Not all of the data are publicly available because they contain information that could compromise children's privacy and family consent.

## Results

### Clinical Findings

The demographic and clinical features of our patients are summarized in table 1. Extensive clinical details are available in the supplemental data (doi.org/10.5061/dryad.w0vt4b8qr). All but 2 individuals (II:3 from family 5, II:1 from family 9) were males. All affected individuals had focal epilepsy with impaired awareness triggered by the experience of bathing or showering, regardless of water temperature. Seizures were typically triggered by pouring water over the head and consisted of behavioral arrest associated with pallor, cyanotic lips, buccal automatisms, and transient hypotonia (supplemental data and videos 1-5). Evolution to bilateral tonic-clonic seizures was clearly described in 4, and loss of consciousness was reported in 1 individual. In 2 individuals, seizures were also triggered by rubbing with a towel after showering (supplemental data, video 1). Seizures also occurred in 1 individual while washing hands and during the immersion of feet in the water, including seawater (II:1, family 1). Moreover, 2 patients also experienced a bilateral tonic-clonic seizure while watching their relatives take a shower or by the thought of bathing/showering (II:1, family 1; II:1, family 6). One adult (IV:2, family 10) had an improvement in seizure control after predominantly showering rather than bathing in warm water. Additional triggers were fingernail clipping in 2 individuals (II:1, family 6; II:1, family 8), 1 of whom also experienced seizures provoked by haircutting (II:1, family 6). The age at onset of provoked seizure ranged from 8 months to 15 years, with weekly to monthly frequency.

10.1212/012298_Video_1 Video 1 Video of case II:1 of family 1. Case II:1 presenting a reflex seizure at the age of 6 years triggered by rubbing with a towel after showering. The episode was characterized by impairment awareness, pallor, buccal automatism, and losing of axial tone lasting less than a minute.Download Supplementary Video 1 via http://dx.doi.org/10.1212/012298_Video_1

10.1212/012298_Video_2 Video 2 Video of case II:1 of family 2. Case II:1 presenting a reflex seizure at age of 1 year during bathing, characterized by impaired awareness, eye blinking, and transient hypotonia.Download Supplementary Video 2 via http://dx.doi.org/10.1212/012298_Video_2

10.1212/012298_Video_3 Video 3 Video of case II:1 of family 3. Case II:1 presenting a reflex seizure at age of 8, characterized by impaired awareness, losing of axial tone, up-rolling eye movements, and lip cyanosis.Download Supplementary Video 3 via http://dx.doi.org/10.1212/012298_Video_3

10.1212/012298_Video_4 Video 4 Video of case II:1 of family 6. Ictal video EEG recording during a bath in participant II:1. A focal dyscognitive seizure starts (00:08) with staring and left head deviation followed by oral automatisms, pallor, and loss of consciousness. The seizure lasts >3 minutes, and ictal EEG shows an initial theta high-voltage polymorphic activity over the frontal temporal region. During the course of the seizure, the EEG recording shows a predominant high-voltage delta activity over the posterior (right > left) brain areas, and it ends (03:40) with a slowed activity over the posterior right hemisphere. ECG also noted a prolongation of the QT interval during the seizure.Download Supplementary Video 4 via http://dx.doi.org/10.1212/012298_Video_4

10.1212/012298_Video_5 Video 5 Video of case II:1 of family 7. Ictal video EEG recording during and after a bath in participant II:1. A focal dyscognitive seizure starts after bathing (01:12) with staring, head deviation to the right, oral automatism, and pallor. Ictal-video EEG shows high-voltage polymorphic theta activity over the right frontal-temporal regions.Download Supplementary Video 5 via http://dx.doi.org/10.1212/012298_Video_5

**Table 1:**
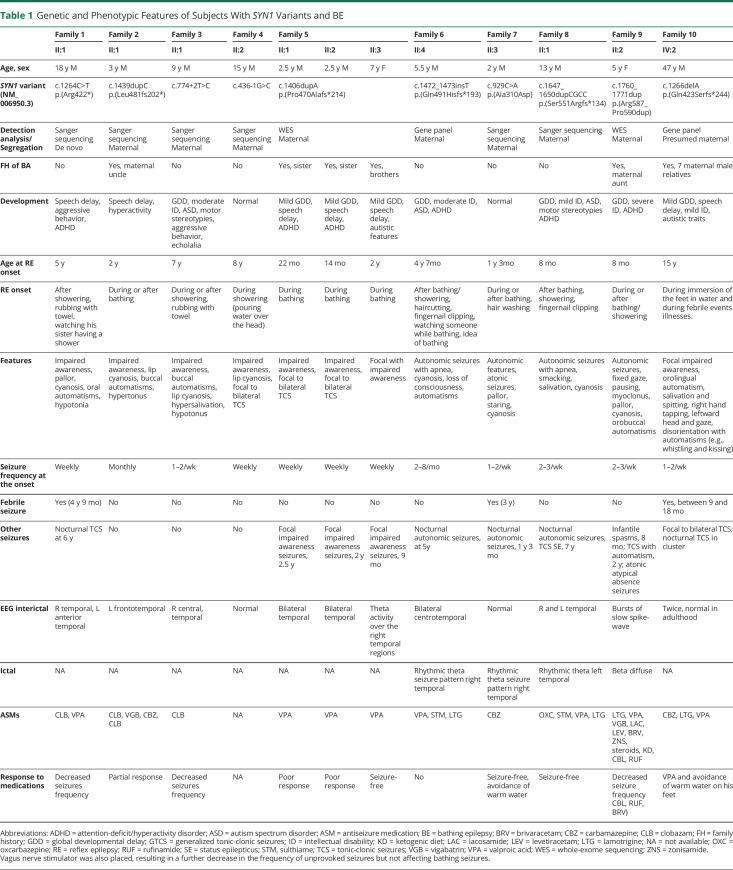
Genetic and Phenotypic Features of Subjects With *SYN1* Variants and BE

Nine participants also developed unprovoked seizures, including focal to bilateral tonic-clonic nocturnal seizures with autonomic features in 5 individuals. Three individuals reported febrile seizures, occurring before the onset of provoked seizures in 2 of them. One additional individual (II:3, family 5) had no provoked focal seizures before the onset of BE.

All individuals received antiseizure medications. Half of them had a satisfactory response mainly to clobazam or valproic acid, and 3 achieved complete control of seizures.

**Figure 2:**
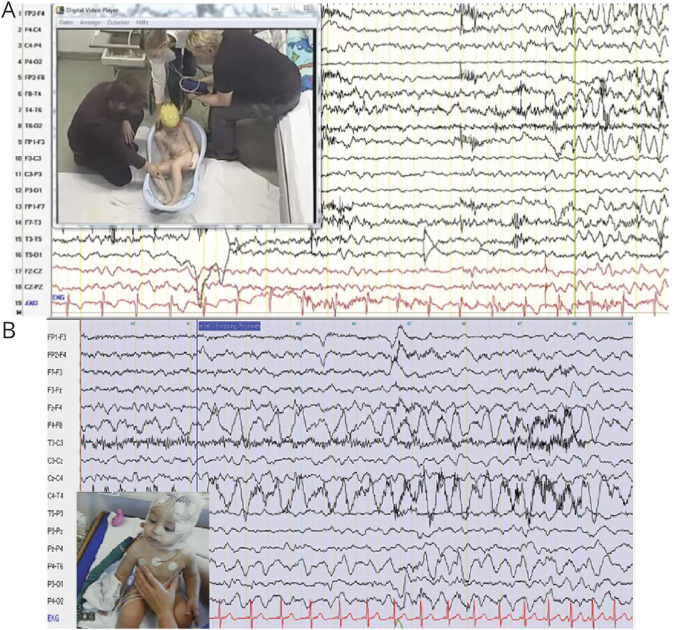
Ictal EEG in 2 Patients With *SYN1* (A) Ictal-video EEG (after bathing) of participant II:4 of family 6 showing the onset of seizure with initial theta high-voltage polymorphic activity over the frontal-temporal region. (B) Ictal-video EEG (after bathing) of participant II:3 of family 7 showing high-voltage polymorphic theta activity over the right frontal-temporal area.

Ictal EEG recording showed high-voltage polymorphic theta activity over the frontal-temporal areas in 2 participants (figure 2). Interictal findings in other participants are available in the supplemental data (figures e-1 and e-2, doi.org/10.5061/dryad.w0vt4b8qr). Brain MRI was performed in 10 of the 13 participants with unremarkable findings.

All but 2 participants (II:1, family 4; II:1, family 7) had a delay and cognitive impairment. Specifically, 8 individuals had a global developmental delay (GDD), and 5 of them were found to have intellectual disability that ranged from mild to severe. Of note, 6 participants were diagnosed with attention-deficit/hyperactivity disorder, and 1 individual had hyperactivity. Three participants had ASD. Behavioral issues such as aggressive behavior were noticed in 2 participants. One adult (IV:2, family 10) now lives and works with minimal support. The clinical features of 21 patients with HWE are summarized in table e-1 (doi.org/10.5061/dryad.w0vt4b8qr). Twenty participants had focal seizures, 8 of whom also developed focal to bilateral seizures. Unprovoked seizures occurred in 62% of cases. Seizure activity was recorded mainly over unilateral temporal regions. Seizure control was achieved by reducing the temperature and duration of the bath or shower and by antiseizure medications such as carbamazepine.^10^

### Genetic Findings

We identified 8 distinct pathogenic variants in *SYN1* (NM_006950.3)c.1264C>T p.(Arg422*) in case II:1 of family 1; c.1439dupC p.(Leu481fs202*) in case III:1 of family 2; c.774 +2T>C in case II:1 of family 3; c.436 -1G>C in case II:1 of family 4; c.1406dupA p.(Pro470Alafs*214) in cases II:1, II:2, and II:3 of family 5; c.1472_1473insT p.(Gln491Hisfs*193) in case II:IV of family 6; c.1647_1650dupCGCC p.(Ser551Argfs*134) in case II:1 of family 8; c.1266delA p.(Gln423Serfs*244) in case II:2 of family 10; and 2 variants of unknown significance [c.929C>A p.(Ala310Asp) in case II:3 of family 7; c.1760_1771dup p.(Arg587_Pro590dup) in case II:2 of family 9] (table 1 and figure 1B). All variants were absent in the gnomAD database. The c.1264C>T p.(Arg422*) variant, previously reported,^18^ occurred de novo, while all other variants were novel and maternally inherited or presumed to be maternally inherited in family 10. The c.436-1G>C and c.774+2T>C variants are predicted to severely affect the protein structure through aberrant mRNA splicing. The c.1264C>T p.(Arg422*) is predicted to undergo nonsense-mediated mRNA decay or result in a truncated protein. All the frameshift variants [c.1439dupC p.(Leu481fs202*), c.1406dupA p.(Pro470Alafs*214), c.1472_1473insT p.(Gln491Hisfs*193), c.1647_1650dupCGCC p.(Ser551Argfs*134) and c.1266delA p.(Gln423Serfs*244)] lead to new reading frames predicted to give rise to a truncated or degraded protein. Although the missense c.929C>A p.(Ala310Asp) and the in-frame insertion c.1760_1771dup p.(Arg587_Pro590dup) variants are classified as variants of unknown significance, they are predicted to have a deleterious effect by multiple in silico analysis and evolution conservation tools. The clinical association with BE further supports their likely pathogenic role. Whole-exome sequencing failed to identify additional pathogenic or likely pathogenic variants in any other Online Mendelian Inheritance in Man genes in all tested individuals. No pathogenic or likely pathogenic variants in *SYN1* were identified in the HWE cohort.

## Discussion

In the first original description of *SYN1* family, it was mentioned the occurrence of water-induced seizures in a patient carrying the p.(W356*) *SYN1* variant.^15^ However, since the first report of 7 individuals belonging to the same large French-Canadian family carrying the truncating variant p.(Gln555*),^17^ only 2 additional individuals harboring distinct nonsense p.(Arg422*)^18^ and missense p.(Ile319Thr) variants^19^ in *SYN1* have been described (table e-2, doi.org/10.5061/dryad.w0vt4b8qr). All patients had reflex seizures triggered by bathing or showering and variable neurodevelopmental disorders ranging from dyslexia or specific language impairments to pervasive developmental disorders. Here, we describe the largest cohort of patients with BE carrying *SYN1* mutations.

Apart from the previously reported nonsense c.1264C>T p.(Arg422*) variant,^18^ all other identified alleles are novel and include 2 splicing-site, 5 distinct frameshift, 1 missense, and 1 in-frame insertion variants (figure 1B). The nonsense, frameshift, and splicing variants are predicted to act through a loss-of-function mechanism like most *SYN1* mutations linked to BE. Although we did not functionally assess the impact of the missense and in-frame variants, they affect the highly conserved residue of the protein and are located in important functional domains. Overall, *SYN1* variants related to BE are clustered in the Pro-rich regulatory domain, while the few variants not associated with BE are also found in other protein domains. However, given the limited number of individuals reported so far, further studies are needed to corroborate this observation and to provide further insights into the genotype-phenotype correlations (figure 1B). Moreover, we observed intrafamilial variability as pointed out by the segregation analysis in family 2 in which the maternal uncle displayed unprovoked seizure and ASD but not BE. This is in line with the previous evidence^17^ suggesting that all individuals harboring SYN1 mutations have variable neurodevelopmental impairments yet not all develop BE.

The main clinical features of our cohort are consistent with the core phenotype of BE (table 1). All but 1 individual presented with seizures provoked by showering or bathing regardless of water temperature. One individual experienced recurrent seizures provoked by immersion of feet in water, not by pouring of water over the head. Additional triggers were rubbing with a wet towel, fingernail cutting, and haircutting in some individuals. In 2 individuals, seizures were also provoked by watching someone taking a bath or just thinking about having a bath.

Of note, we report the first 2 female individuals with BE. They also displayed a variable degree of developmental delay, from mild GDD to severe cognitive impairment associated with unprovoked seizures. We hypothesize that skewed X-inactivation occurring in the brain tissues could explain the expression of the disease in these female carriers.

Nine of the 13 participants also developed unprovoked seizures. In 2 of them, febrile seizures preceded the onset of bathing seizures. Other unprovoked seizures occurred at night in 5 participants and were mostly focal or focal to bilateral with autonomic features. Only 1 individual had a prolonged seizure resulting in status epilepticus.

Antiseizure treatment was required in all participants, and partial or complete control of seizures was achieved in 6 cases. Clobazam and valproic acid were the most effective drugs. Ictal EEG recorded in 2 participants showed rhythmic theta activity over the frontocentral/temporal regions, in keeping with the previous reports.^9,17^ Variable cognitive impairment was noticed in 10 of 13 participants. ASD, attention-deficit/hyperactivity disorder, and behavioral issues were also predominant features in several participants.

Overall, the clinical and electrophysiologic findings in our patients overlap those described in previous BE cases, suggesting that this condition is a specific and preventable RE related to contact with water.

*SYN1* encodes a neuron-specific phosphoprotein implicated in the regulation of neurotransmitter release and synaptogenesis.^22^ Its role in epilepsy has been elucidated by studies in the *SYN1* knockout mouse model showing impaired synaptic vesicle trafficking and impairment of GABA release through a loss-of-function mechanism that results in higher network excitability and firing activity.^22,23^ Although the exact pathophysiology of *SYN1*-related BE is currently unknown, the ictal SPECT findings in some individuals have suggested insular cortex involvement.^17^ Indeed, the insula is a key integrative multisensory area, well connected with the temporal lobe^24^ and potentially able to generate motor and autonomic symptoms, like those observed in BE cases, if functionally perturbed by genetic mutations.^17^ Accordingly, *SYN1* mutations may lead to imbalances between excitatory and inhibitory influences at the synaptic level, thus entraining temporal and insular areas into a seizure activity after water pouring.^17^

REs represent a spectrum of conditions characterized by broad clinical and genetic heterogeneity and several overlapping features.^1,6^ Apart from *SYN1*, a few additional REs-related genes have been reported, including *SYNGAP1* in individuals with chewing reflex,^25,26^ *CDKL5*-related disorders exhibiting diaper change reflex,^27^ *SCN1A* in somatosensory reflex seizures,^28^ and *CHD2* in photosensitive seizures.^5^ It is likely that in all these epilepsy types, the genetic defect eventually results in abnormal hyperexcitability of cortical areas that are physiologically activated during specific sensory stimulation, acting as triggers.

The recent report of HWE in an individual carrying a splice variant (c.527+1G>T) in *SYN1* may argue whether BE and HWE belong to the same phenotypic spectrum.^20^ Thus, to specifically address this issue, we screened a cohort of cases showing HWE and found no evidence of pathogenic variants in *SYN1*.

HWE, largely reported in southern India, is induced by bathing with hot water usually >37°C.^29^ Seizures often occur when individuals are seated and hot water is poured from a washtub or basin over their heads.^10,29^ Seizures may also start with self-induction in some patients as they enjoy this situation. Similar to BE, there is a male-to-female predominance, and ≈20% to 40% of individuals with HWE may develop spontaneous seizures,^8^ but unlike those with BE, the majority of individuals have normal development. Several studies, including EEG and fMRI, have suggested a predominant temporal lobe involvement, with the possible contribution of parietal and occipital areas.^30,31^ SPECT studies have demonstrated ictal hypermetabolic uptake in the medial temporal structures and hypothalamus.^32^ Although the physiopathology of HWE remains unknown, it has been suggested that it could be related to a hyperthermic kindling involving the thermoregulation center of the hypothalamus that triggers seizures.^33,34^

Despite a similar ictal semiology and EEG, our data suggest that BE and HWE are likely distinct epileptic disorders with different genetics, seizure triggers (pouring vs hot water), and hyperexcitability of cortical circuits. The improvement observed after decreasing water temperature in HWE^33^ and the report of a few individuals with BE who also experienced seizure precipitated by rubbing the face with a wet cloth or nail clipping^17^ further support our thoughts. Similarly, 2 of our patients also experienced seizures triggered by rubbing with a towel after showering. The other 2 individuals had seizures triggered by fingernail clipping and one of them also by haircutting. Taken together, these findings suggest that BE is intrinsically related to a somatosensory stimulus rather than the simple water temperature, as instead observed in HWE. Hence, it may be possible that the reflex seizure reported in 2 *SYN1* cases, apparently after hot water exposure, was instead precipitated by the somatosensory stimulus of water, and the temperature played only a confounding role. Moreover, the report of 2 individuals with seizures provoked by watching someone bathing or showering suggests that the pathophysiology of *SYN1*-related RE could be even more complex, likely involving mirror-like activities and yet unknown and tightly regulated neuronal circuits.

Recent simulation theories in cognitive neuroscience emphasize that sensorimotor capacities and cognitive processes are inseparable because the simulation process involves the same sensorimotor neural correlates that are active during the action execution or interaction with the actual object or entity itself.^35^ Accordingly, watching someone else bathing or showering or imagining bathing or showering may involve the same neuronal circuits that trigger the seizure when acting.

BE is a clinically and genetically homogeneous distinct RE and should be considered a handle for the molecular diagnosis of *SYN1*-related epilepsy. Early identification of the molecular defect may help start early intervention strategies to optimize function and quality of life and to prevent comorbid conditions in affected patients. Future studies using advances in electrophysiology and imaging data acquisition will help to define the genotypic-phenotypic spectrum and understand the underlying pathomechanisms of this rare RE to eventually develop effective and targeted therapeutic strategies.

## Glossary

ASD: autism spectrum disorder
BE: bathing epilepsy
GDD: global developmental delay
HWE: hot-water epilepsy
RE: reflex epilepsy
SYN: Synapsin
